# Electron Beam Irradiation-Induced Defects Enhance Pt-TiO_2_ Photothermal Catalytic Degradation in PAEs: A Performance and Mechanism Study

**DOI:** 10.3390/molecules30030697

**Published:** 2025-02-05

**Authors:** Fukun Bi, Yaofei Zhang, Zhuoxuan Zhou, Lei Guo, Ziqiao Zhu, Baolin Liu, Xiaodong Zhang

**Affiliations:** 1 School of Environment and Architecture, University of Shanghai for Science and Technology, Shanghai 200093, China; bifukun@usst.edu.cn (F.B.); 15517828571@163.com (Y.Z.); zzx200011060317@163.com (Z.Z.); zhuziqiao229@163.com (Z.Z.); 2 School of Health Science and Engineering, University of Shanghai for Science and Technology, Shanghai 200093, China; blliuk@163.com; 3 School of Materials and Chemistry, University of Shanghai for Science and Technology, Shanghai 200093, China; 17886448863@163.com; 4 Shanghai Non-Carbon Energy Conversion and Utilization Institute, Shanghai 200240, China

**Keywords:** SVOCs, TiO_2_, photothermal catalysis, electron beam irradiation, defect

## Abstract

Phthalic acid esters (PAEs), ubiquitous semi-volatile organic compounds (SVOCs) in indoor environments, pose adverse effects on human health. However, their degradation mechanisms and pathways remain unclear. Herein, we developed an efficient photothermal catalyst by introducing defects (oxygen vacancies, O_V_s) on TiO_2_ (P25) surfaces via electron beam irradiation technology with different irradiation doses (100, 300, 500, and 700 kGy). The TiO_2_ with defects was employed as a support to prepare Pt-TiO_2_ catalysts for the photothermal degradation of di (2-ethylhexyl) phthalate (DEMP) and dimethyl phthalate (DMP), two representative PAEs. TiO_2_ pre-treated with a 300 kGy irradiation dose supported the Pt catalyst (Pt-Ti-P-300) and presented the optimal catalytic performance for DEMP and DMP degradation. Characterization results confirmed that O_V_s were successfully introduced to the catalysts. Meanwhile, O_V_s induced by electron beam irradiation expanded the light absorption range and improved the generation and separation of photogenerated carriers, which significantly enhanced the catalytic activity of the catalysts for PAE degradation. Importantly, the degradation mechanism and pathway of DMP were further explored by using in situ diffuse reflectance infrared Fourier transform spectroscopy (DRIFTS) and gas chromatography–mass spectrometry (GC-MS). These findings provide important insights into the electron beam irradiation-mediated regulation of catalysts and the photothermal catalytic removal of PAEs in indoor environments.

## 1. Introduction

Defects in metal oxides play an important role in modulating catalytic properties [1,2,3]. Surface defects in materials can promote the adsorption and activation of substrate molecules and contribute positively to reaction thermodynamics and kinetics [4]. To obtain superior catalytic properties, defect engineering based on metal oxides has been extensively studied in the last decades [2,5,6]. In metal oxides, the generation of defects is inevitable and can result in the loss of translational symmetry in the crystal cell [1]. Defects caused by such symmetry breaking can occur in multiple dimensions, resulting in three-dimensional bulk defects, two-dimensional planar defects, one-dimensional linear defects, and zero-dimensional point defects [7,8,9]. All these defects can lead to changes in metal oxides’ electronic properties [10,11]. Among these defects, zero-dimensional point defect oxygen vacancies (O_V_s), as intrinsic defects, greatly influence the optimization of catalytic performance [12].

In photocatalytic systems, O_V_s can be considered to play an active role as a type of surface defect [13,14]. In essence, O_V_s facilitate the photocatalytic degradation of volatile organic compounds (VOCs) by establishing an electric field in metal oxide semiconductors, thereby enhancing light absorption and charge transfer. A pivotal stage in the thermocatalytic oxidation of VOCs is the activation of molecular oxygen [15,16,17]. The activation process of molecular oxygen and the type of active oxygen produced are dependent on the surface defects. The abundance of O_V_s in catalysts facilitates the activation of molecular oxygen and the generation of reactive oxygen species [18]. These activated oxygen species react rapidly with the adsorbed substrates, which are then converted to intermediates or completely mineralized, leading to the degradation of target pollutants. Consequently, photothermal catalysis unites the distinctive benefits of O_V_s in photocatalysis and thermal catalysis, representing a highly promising technology for the treatment of VOCs [19,20,21]. Prior research has demonstrated that the equilibrium concentration of O_V_s in metal oxides is relatively low. However, certain treatments have been shown to significantly increase their concentration [22]. For instance, chemical reduction, high-temperature treatment, and electron beam irradiation (EBI) represent effective techniques for increasing O_V_s [23]. Among these techniques, EBI technology shows the greatest potential for future development. In comparison to alternative treatment technologies, EBI does not necessitate the introduction of additional chemical reagents [24,25]. Furthermore, the irradiation dose can be meticulously calibrated at relatively low temperatures, thereby enabling the precise regulation of the concentration of O_V_s. For example, Ding et al. [26] used EBI with an energy of 0.1 MeV to modify the surface structure of TiO_2_ nanorod arrays (NRs). The photoelectrochemical (PEC) water decomposition activity of the EBI-TiO_2_ photoanode was greatly enhanced due to the introduction of enriched O_V_s on the surface of the TiO_2_ NRs by the EBI treatment. Li et al. [27] induced the structural evolution of anatase TiO_2_ to cubic TiO_2_ by electron beam irradiation. The formation of surface O_V_s and the driving of the rearrangement and migration of Ti atoms were demonstrated both theoretically and experimentally.

Phthalic acid esters (PAEs) are environmental endocrine disruptors and derivatives of phthalic acid, which is generally used in industrial production as a softener and plasticizer for plastics [28]. The content of PAEs in plastic products ranges from 20% to 30%, and they are prone to migrating into the environment because they are not fully polymerized with the plastic matrix. DMP and DEMP are representative phthalate esters (PAEs) that are widely used in plastic products, cosmetics, and detergents, and have adverse influences on human fertility and the endocrine system [29]. In our previous study, a commercial P25-supported Pt catalyst exhibited significant photothermal catalytic activity for DEMP. Thus, it is also necessary to investigate the applicability of catalysts to this class of pollutants.

In this work, the electron beam irradiation technique was used to treat commercial P25 to introduce further O_V_s in the structure. Subsequently, the irradiated P25 was selected as the support to prepare supported Pt catalysts by conventional wet impregnation, and their photothermal catalytic activity was investigated via DEMP and DMP degradation. The influence of EBI on the catalyst was analyzed by many characterizations to clarify the generation and evolution mechanism of O_V_s, and the optimal irradiation dose was screened. Importantly, the degradation mechanism and pathway of DMP were explored by in situ diffuse reflectance infrared Fourier transform spectroscopy (DRIFTS) and gas chromatography–mass spectrometry (GC-MS).

## 2. Results and Discussion

### 2.1. Structure Characterization

**Figure 1:**
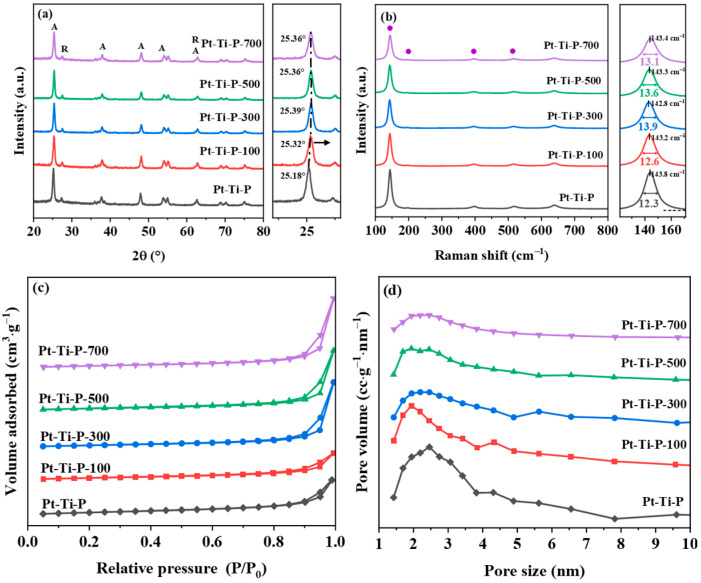
(**a**) XRD, (**b**) Raman patterns, (**c**) N_2_ adsorption and desorption isotherms, and (**d**) pore size distribution curves for the Pt-Ti-P and Pt-Ti-P-x catalysts.

The catalysts Pt-Ti-P and Pt-Ti-P-x (x = 100, 300, 500, and 700, representing the irradiation doses) were prepared via the impregnation method by using TiO_2_ (P25) and its irradiated samples with different electron beam irradiation doses as the supports. X-ray diffraction (XRD) was performed to determine the crystalline phase of the catalysts. As displayed in Figure 1a, the diffraction peaks of Pt-Ti-P-x showed a mixed crystalline pattern of anatase and rutile phases [30]. This indicated that the catalysts maintained the crystal structure of P25 after EBI. The absence of diffraction peaks of Pt and PtO_x_ at 39.76° (JCPDS 65-2868) and 34.86° (JCPDS 37-1087) may be attributed to the generation of fine platinum clusters or single atoms of Pt on Pt-Ti-P-x, which highly dispersed on the surface of the catalyst. After irradiation treatment, the diffraction peak intensity of Pt-Ti-P-x decreased. This reduction in characteristic peak intensity may be caused by structural defects introduced by radiation. Generally, the diffraction peak intensity is closely connected with the degree of crystallinity and the ordering of the crystal structure. Higher peak intensity typically indicates a more ordered arrangement of crystals in the sample. In contrast, the presence of structural defects disrupts the crystal order, resulting in a decrease in peak intensity [31]. Additionally, the P25 characteristic peaks of Pt-Ti-P-x were significantly shifted. Compared with P25 (25.28°) in our previous work [14] and Pt-Ti-P (25.18°), the P25 characteristic peaks of Pt-Ti-P-x (25.32~25.39°) are all shifted to high angles, with Pt-Ti-P-300 exhibiting the largest shift. The shift of the characteristic peaks to higher angles in Pt-Ti-P-x indicated an increase in the lattice constant, which is typically associated with lattice distortion. This distortion is often indicative of the formation of point defects and is directly related to the presence of O_V_s [26,32]. Raman spectroscopy (Figure 1b) was used to further explore the catalysts’ structure. Raman bands observed at 151 cm^−1^ (E_g_), 199 cm^−1^ (E_g_), 393 cm^−1^ (B_1g_), 515 cm^−1^ (B_1g_/A_1g_), and 635 cm^−1^ (E_g_) belong to the anatase TiO_2_ [33,34]. Compared with Pt-Ti-P, the characteristic TiO_2_ in Pt-Ti-P-x exhibited a significant intensity reduction, indicating that the introduction of irradiation induced lattice distortion in the catalyst, which is consistent with the XRD results. Compared to the Raman spectra positions of Pt-Ti-P, the Raman spectra positions of Pt-Ti-P-x were shifted to lower wave numbers by 0.4~1 cm^−1^. According to Hooke’s law, the Ti-O bond strength was positively correlated with the Raman spectra position, indicating that the introduction of electron beam irradiation weakened the Ti-O bond strength in the catalysts and introduced defects [35]. Furthermore, the full-width half maximum (FWHM) was computed through Lorentz and Gauss fitting curves. The FWHM of Pt-Ti-P-x increased significantly, with varying degrees of increment. It has been reported that the concentration of O_V_s is correlated positively with Raman FWHM values [36,37]. Figure 1b shows the FWHM values of the samples, where Pt-Ti-P-300 (13.9) exhibits the largest increase in FWHM values compared to Pt-Ti-P (12.3), suggesting that Pt-Ti-P-300 may contain the highest concentration of O_V_s. The pore structure of catalysts affects the distribution of active sites and the mass transfer of reactant molecules. N_2_ adsorption–desorption curves and pore diameter distribution profiles of Pt-Ti-P-x are presented in Figure 1c,d. All samples showed type III curves with H3 hysteresis loops, suggesting the existence of irregular mesoporous structures. The mesoporous structure facilitates molecular mass transfer in catalytic reactions [38]. The differences in specific surface areas (Table 1) between P-Ti-P (52.0 m^2^/g), Pt-Ti-P-100 (55.9 m^2^/g), Pt-Ti-P-300 (51.9 m^2^/g), Pt-Ti-P-500 (54.2 m^2^/g), and Pt-Ti-P-700 (55.4 m^2^/g) were not significant, suggesting that the surface area did not affect the catalytic performance. The above results indicate that EBI induces lattice distortion in the catalyst and promotes the formation of defects within the catalyst.

### 2.2. Oxygen Vacancy Identification and Electronic Structure Evaluation

**Figure 2:**
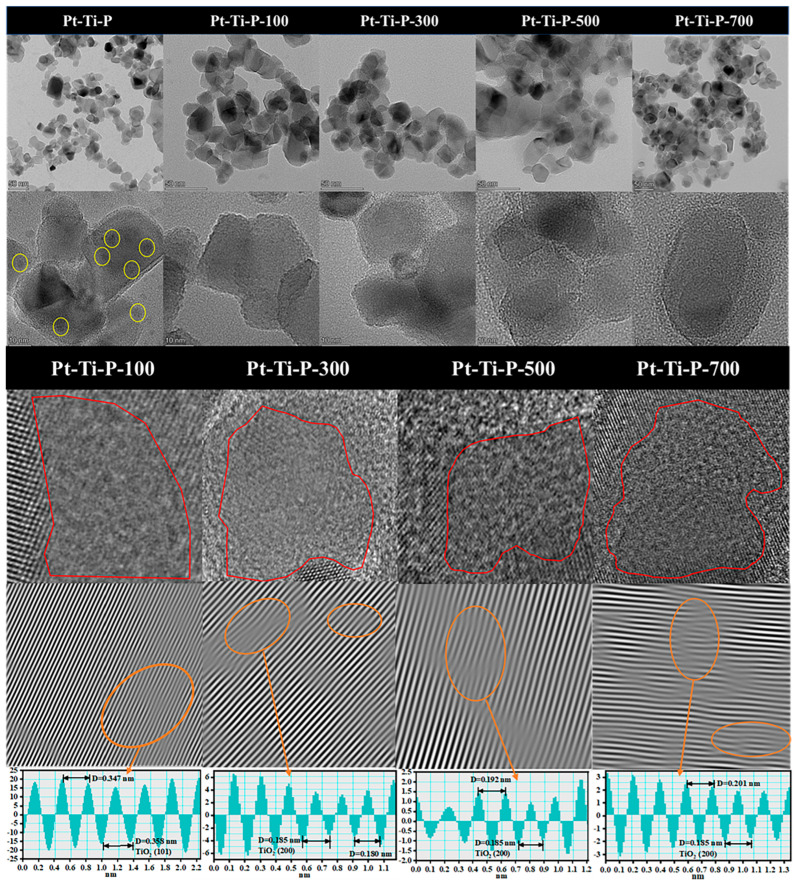
TEM, HRTEM, and FFT patterns of Pt-Ti-P and Pt-Ti-P-x.

**Figure 3:**
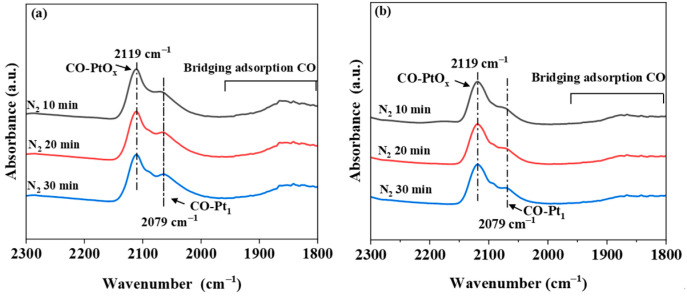
DRIFTS spectrogram of the CO in situ adsorption of (**a**) Pt-Ti-P and (**b**) Pt-Ti-P-300 (after 30 min of CO adsorption; spectra were collected after N_2_ blowing for 10, 20, and 30 min, respectively).

TEM was applied to study the morphology and lattice structure. All samples presented irregular polyhedral structures (Figure 2). In contrast to the clear Pt particles (yellow circles) on the Pt-Ti-P surface, no Pt particles were observed on the Pt-Ti-P-x surface, suggesting that Pt was highly dispersed on the P25 surface. HRTEM was used to further observe the defects caused by EBI on the surface of the catalysts. Compared with Pt-Ti-P, some regions of the rough surface (red circles) of the Pt-Ti-P-x catalyst showed dark pits and structural distortions, which showed strong disorder and distortion of the lattice stripes. The Pt species on the catalyst surface before and after irradiation were further investigated by using in situ DRIFTS CO adsorption. Figure 3 presents the in situ DRIFTS CO adsorption spectra over Pt-Ti-P (Figure 3a) and Pt-Ti-P-300 (Figure 3b). Three bands at 2119, 2079, and 1860 cm^−1^ were found on all samples. The band at 2119 cm^−1^ is attributed to the linear adsorbed CO on oxidized Pt species (CO-PtO_x_) [39]. The band at 2079 cm^−1^ is associated with CO adsorption on single Pt atoms (CO-Pt_1_) [40]. The band at 1860 cm^−1^ corresponds to the bridged CO adsorption on Pt NPs [40]. The broad band (1860 cm^−1^) corresponding to Pt NPs in Pt-Ti-P-300 exhibits a significant decrease in intensity, indicating that irradiation further enhances the dispersion of Pt on the P25 surface.

**Figure 4:**
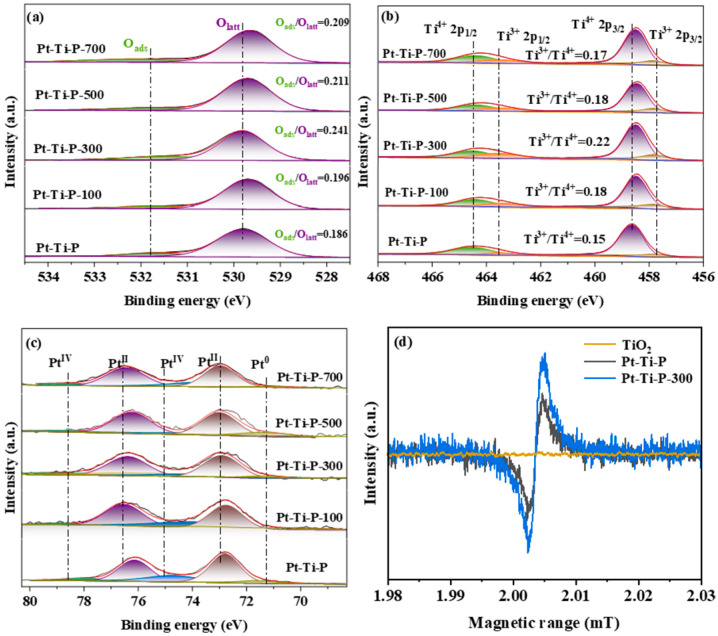
XPS spectra of Pt-Ti-P and Pt-Ti-P-x: (**a**) O 1s, (**b**) Ti 2p, and (**c**) Pt 4f; (**d**) EPR spectra of TiO_2_ and the Pt-Ti-P and Pt-Ti-P-300 catalysts.

Chemical states on the catalyst surface were studied by XPS, because the O_V_s can influence the coordination of O, Ti, and Pt atoms [41,42]. As depicted in Figure 4a, the asymmetrical spectra of O 1s were fitted into lattice oxygen (O_latt_, 529.7~529.8 eV) and adsorbed oxygen (O_ads_, 531.5~532.2 eV), respectively [43]. The O_ads_/O_latt_ ratios were calculated from the fitted spectra areas and are listed in Table 1. Compared with Pt-Ti-P, the O_ads_/O_latt_ ratio is substantially higher with Pt-Ti-P-x. This suggests that EBI effectively introduces O_V_s into the catalyst, and that an irradiation dose of 300 is optimal. For Ti 2p (Figure 4b), the asymmetrical spectra were fitted into Ti^4+^ 2p_1/2_ (464.8 eV), Ti^3+^ 2p_1/2_ (463.7 eV), Ti^4+^ 2p_3/2_ (459.0 eV), and Ti^3+^ 2p_3/2_ (458.3 eV) [44,45]. After the irradiation treatment, the Ti^3+^/Ti^4+^ ratio in Pt-Ti-P-x increased significantly. The introduction of Ti^3+^ induced changes in the lattice structure of P25. The formation of Ti^3+^ tends to create O_V_s to maintain electrostatic equilibrium according to the chemical Equation (1):

4Ti^4+^ + O_2_ → 2Ti^4+^ + 2Ti^3+^ + Ov + 0.5O_2_ (1)

Due to the creation of a charge imbalance, some lattice oxygen was activated or removed from the P25 lattice, generating O_V_s. This is consistent with the results from the O 1s spectra [46]. In addition, compared to Pt-Ti, the Ti 2p binding energy of Pt-Ti-P-x is reduced after irradiation, indicating an increase in the electron cloud density. Pt^2+^ (72.1–72.5 eV and 75.6–75.9 eV), and Pt^4+^ (75.6–75.9 eV) are mainly present in Pt 4f (Figure 4c). The molar ratio of active center PtO_x_ to Pt_total_ (PtO_x_/Pt_total_) was calculated according to the peak areas of Pt (Table 1). Pt-Ti-P-300 exhibits the highest PtO_x_/Pt_total_ molar ratio. Importantly, compared to Pt-Ti-P, the Ti 2p binding energy of Pt-Ti-P-x is increased after irradiation, indicating a decrease in the electron cloud density. This suggests that the defects introduced by electron irradiation promote electron transfer between Pt and TiO_2_.

To further verify the existence of O_V_s, EPR tests were conducted on TiO_2_, Pt-Ti-P, and Pt-Ti-P-300 at room temperature under dark conditions. As presented in Figure 4d, no corresponding characteristic peaks were detected on TiO_2_, whereas there is a distinct peak at g = 2.002 for both Pt-Ti-P and Pt-Ti-P-300, which is attributed to the formation of peaks by single-electron capture of O_V_s [47]. Moreover, the peak intensity of irradiated Pt-Ti-P-300 is larger, which is proportional to the concentration of O_V_s. Therefore, the EPR results show that O_V_s were generated on the surface of P25 after irradiation with the high-energy electron beam, which could provide more active sites for the catalytic degradation reaction, increasing reaction efficiency. The above results indicate that the lattice distortion induced by irradiation leads to an increase in Ti^3+^ content and the introduction of O_V_s. The presence of Ti^3+^ and O_V_s facilitates the deep oxidation of pollutants, thereby enhancing photothermal catalytic activity.

### 2.3. Detection of Optical Properties

**Figure 5:**
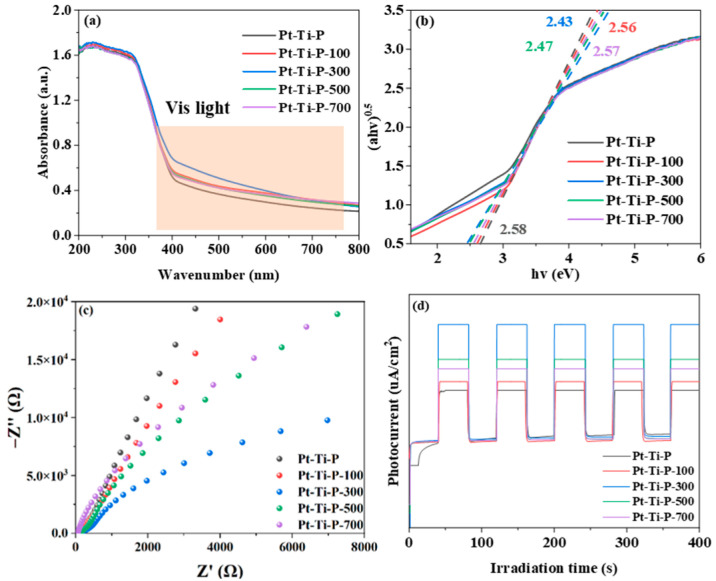
(**a**) UV-vis DRS, (**b**) band gap width, (**c**) EIS, and (**d**) a transient photocurrent plots diagram of Pt-Ti-P and Pt-Ti-P-x.

In previous studies, the optical properties of materials were affected by O_V_s [48]. Compared with Pt-Ti-P, Pt-Ti-P-x exhibits enhanced light absorption in the visible range (380–780 nm), which indicates that the introduction of irradiation improved its light-response abilities (Figure 5a). The UV-vis DRS was processed to obtain a taut plot curve (Figure 5b) of the catalysts. The forbidden bandwidth of Pt-Ti-P-x (2.471~2.609 eV) was significantly narrower than that of Pt-Ti-P (2.673 eV), with Pt-Ti-P-300 (2.471 eV) having the narrowest band gap. In addition, electrochemical impedance spectrum (EIS) and transient photocurrent response (IT) analyses were used to investigate the effect of O_V_s on the charge transfer dynamics in the catalysts. The electrochemical impedance spectra are shown in Figure 5c. All photoelectrodes showed stable photo responses over five cycles. The electrochemical impedance of Pt-Ti-P-x decreased compared with Pt-Ti-P, and Pt-Ti-P-300 showed the lowest electrochemical impedance. The transient photocurrent response is shown in Figure 5d. The transient photocurrent intensities of Pt-Ti-P-x were all enhanced compared with Pt-Ti-P, and Pt-Ti-P-300 showed the highest transient photocurrent intensity strength. The above results indicate that the O_V_s introduced by irradiation promoted the generation, separation, and transfer of photogenerated carriers in the catalysts and greatly improved the charge transfer dynamics.

### 2.4. Catalytic Activity

**Figure 6:**
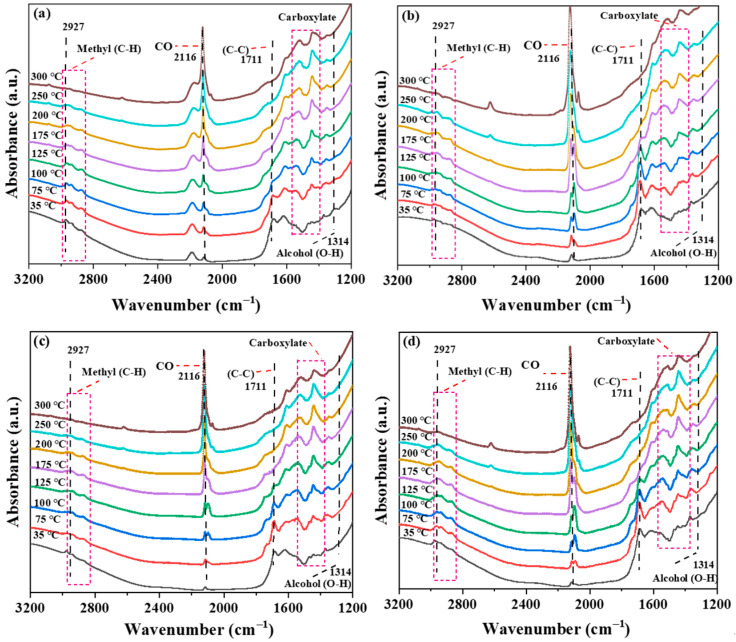
In situ infrared spectroscopy of the photothermal degradation of DEMP by (**a**) Pt-Ti-P-100, (**b**) Pt-Ti-P-300, (**c**) Pt-Ti-P-500, and (**d**) Pt-Ti-P-700.

**Figure 7:**
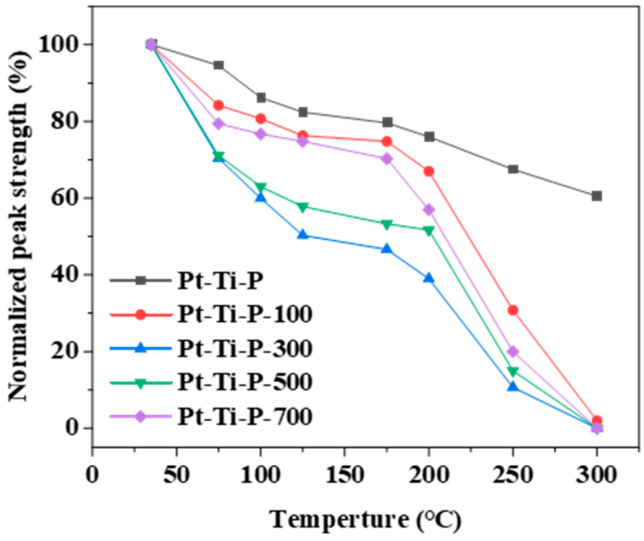
Normalized peak strength changes for methyl.

To further investigate the key role of O_V_s generated by EBI on the catalytic properties of TiO_2_-based composites, the photothermal catalytic properties of samples with different irradiation dosages were tested in a homemade in situ DRIFTS reactor using DEMP as a probe. The in situ DRIFTS spectra are depicted in Figure 6, and multiple typical peaks of DEMP at 2927 cm^−1^ (C-H stretching vibration, methyl), 1711 cm^−1^ (C-C stretching vibration, sp^2^ hybridized carbon), and 1314 cm^−1^ (O-H bending vibrations, alcohols) were observed at 35 °C [49,50,51]. These distinct characteristic peaks indicate the successful adsorption of DEMP on the catalyst surface. As the reaction proceeded, the intensity of these peaks (1314, 1711, and 2927 cm^−1^) gradually decreased with the gradual increase in temperature, and completely disappeared at a temperature of 300 °C, indicating that the DEMP was completely transformed. The peak at 1399~1580 cm^−1^ is assigned to the carboxylate O-C-O asymmetry and symmetry vibrations, and 2116 cm^−1^ is attributed to the carbon monoxide (CO) [52,53,54]. The appearance of the characteristic peaks of carboxylate and carbon monoxide indicates that DEMP was converted to intermediates such as CO and carboxylate during the reaction process. To visualize the effect of O_V_s generated by different irradiation doses on the catalytic performance, the changes in DEMP characteristic peak intensities in the different Pt-Ti-P-x catalysts were compared (Figure 7). As the reaction proceeded, compared with Pt-Ti-P, the methyl peak intensity of the Pt-Ti-P-x showed a significant reduction. Among them, the most obvious diminution was observed for Pt-Ti-P-300. Compared with Pt-Ti-P, the characteristic peak intensity of carbon monoxide for Pt-Ti-P-x showed a significant enhancement, indicating that the O_V_s introduced by electron beam irradiation promoted the conversion of DEMP to intermediates such as CO. The above data indicate that the O_V_s introduced by the EBI technique significantly enhanced the photothermal catalytic performance of the catalysts, and that an irradiation dose of 300 kGy is the optimal irradiation dose for commercial P25.

**Figure 8:**
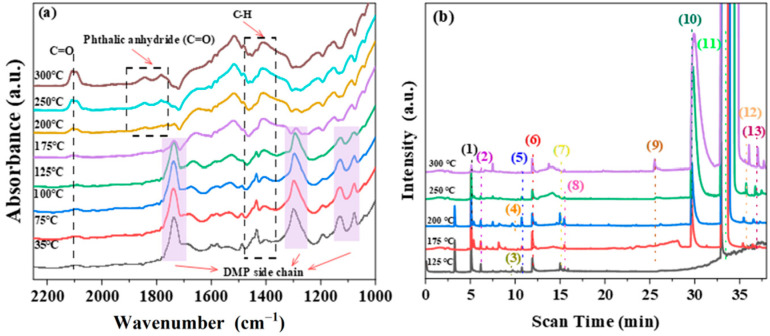
(**a**) In situ infrared spectroscopy of Pt-Ti-P-300’s photothermal catalytic degradation of DMP; (**b**) GC-MS spectra of DMP on Pt-Ti-P-300 at different temperatures of photothermal catalytic oxidation.

To verify the universality of Pt-Ti-P-300 for PAE degradation, the screened oxygen-rich vacancy catalyst was used to perform photothermal catalytic degradation experiments of DMP. As shown in Figure 8a, multiple characteristic peaks of DMP were observed at 35 °C. The peaks at 1580 cm^−1^ and 1490 cm^−1^ could be ascribed to the aromatic ring skeleton stretching vibrations [55,56]. Meanwhile, the characteristic peaks of DMP side chains were clearly observed. The band at 1730 cm^−1^ was assigned to the C=O stretching pattern [57]. The peak at 1293 cm^−1^ was attributed to the C-H stretching pattern [58]. The peaks at 1126 cm^−1^ and 1076 cm^−1^ were attributed to the C-C in-plane deformation vibration and stretching mode of C-O-C, respectively [59]. These distinct characteristic peaks indicate the successful adsorption of DMP onto the catalysts. As the procedure proceeded, the functional groups on the catalyst surface changed significantly. When the reaction temperature was heated to 75 °C, a new absorption band, attributed to the backbone vibration of the benzene ring in phthalic acid, was observed at 1520 cm^−1^ [60]. The intensities of the four characteristic peaks of the DMP side chain were inversely proportional to temperature in the range of 35 °C to 175 °C and disappeared at 175 °C. This indicates that the DMP side chain breaks as the temperature increases. When the temperature further increased to 200 °C, two other peaks were observed at 1778 cm^−1^ and 1842 cm^−1^, which could be the stretching vibrations of the C=O bond in phthalic anhydride [61,62].

### 2.5. Intermediates and Reaction Mechanism

**Figure 9:**
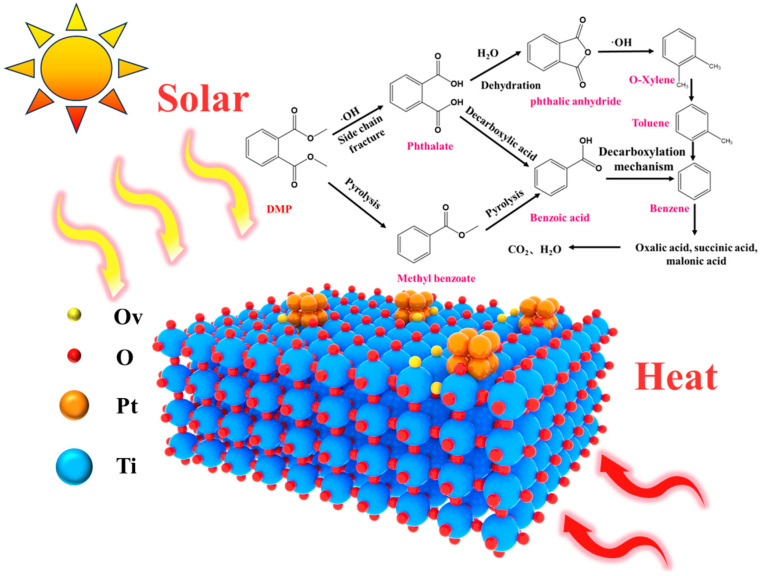
Possible degradation paths of DMP with Pt-Ti-P-300.

The reaction intermediates of the DMP photothermal degradation over Pt-Ti-P-300 were probed by GC-MS, and the degradation pathways were inferred in combination with in situ infrared spectroscopy. Figure 9 shows the GC-MS spectrum of photothermally catalyzed DMP, and the corresponding intermediate product information is summarized in Table 2. Since acetone was used as a solvent in the loading process of the DMP, acetone was detected at all temperature points. The intensity of the DMP peak gradually decreases as the reaction temperature increases. Since no significant intermediate product signals other than phthalic acid were detected below 125 °C, the spectra are shown starting at 125 °C. A strong phthalic acid signal was detected in the reaction off-gas at 125 °C, which gradually increased with the reaction temperature. This suggests that the intermediate product phthalic acid was produced by light exposure, which is consistent with the in situ DRIFTS observations. We also note that methyl benzoate was detected at higher temperatures (175 °C), suggesting that there may be more than one pathway for DMP degradation. In general, photocatalysts produce hydroxyl radicals in the presence of light, and the hydroxyl radicals react with DMP. The hydroxyl radical traps hydrogen atoms from DMP to form the intermediate product phthalic acid [63,64]. This is in agreement with the results of the in situ DRIFTS. However, methyl benzoate was detected at higher temperatures, suggesting that pyrolysis of the DMP side chains also plays a partial role. As the temperature of the reaction increases, light leads to the cleavage of DMP, leading to side chain breaks, which in turn produces methyl formate and methyl benzoate. From this, the possible degradation mechanism of DMP under photothermal exposure was proposed. Under light conditions, Pt-Ti-P-300 generates hydroxyl radicals that react with the methyl group of DMP to form the intermediate product phthalic acid. One part of phthalic acid undergoes dehydration condensation to form phthalic anhydride, and the other part of phthalic acid generates benzoic acid through a decarboxylation mechanism [65]. As the temperature increases, the lattice oxygen in Pt-Ti-P-300 is activated to generate reactive oxygen species, which attack the side chain, leading to side chain breakage and the generation of methyl benzoate. Methyl benzoate continues to crack at high temperatures to produce other by-products, such as benzoic acid.

## 3. Experimental Method

### 3.1. Chemicals and Reagents

Dimethyl phthalate (DMP, C_10_H_10_O_4_, ≥99%) and H_2_PtCl_6_·6H_2_O (≥99%) were bought from Aladdin. TiO_2_ with a 4:1 ratio of anatase to rutile (AEROXIDE P25) was purchased from Hunan Lijie Chemical Co., Ltd., Changsha, China. All materials were used as they were.

### 3.2. Catalyst Preparation

#### 3.2.1. Modification of TiO_2_ by Electron Beam Irradiation

In this experiment, 1.8 MeV high-energy electron beam treatment was used to irradiate the commercial TiO_2_ (P25) powder. The irradiator was a GJ-II electron accelerator (Shanghai Pioneer Electric Factory, Shanghai, China) from the Applied Radiation Institute of Shanghai University, with an adjustable electron beam energy (1.0–2.5 MeV) and beam current (0–20 mA). EBI experiments were performed with an electron beam energy of 1.8 MeV and a beam current of 1.0 mA. Samples weighing 1 g and measuring 1 mm thick were irradiated. The samples were irradiated on a reciprocating moving beam-down device with the sample stage 30 cm from the titanium window. The irradiation doses were 100, 300, 500, and 700 kGy. The TiO_2_ powder treated by EBI was named Ti-P-x (x = 100, 300, 500, and 700, representing the irradiation doses).

#### 3.2.2. Preparation of the Pt-Ti-P and Pt-Ti-P-x Catalysts

The Pt support catalyst was prepared by the wet impregnation method. Firstly, 1 g of irradiated TiO_2_ was poured into a beaker. Then, a certain amount of water (50 mL) and H_2_PtCl_6_·6H_2_O solution (985 μL, 27 mg/mL, theoretical Pt loading: 1.0 wt%) were added and sonicated for 0.5 h at 80 °C. After that, the catalysts were dried at 80 °C in an oven. Finally, the as-prepared catalyst was calcinated at 400 °C in an air atmosphere for 2 h with a heating rate of 10 °C/min. The catalysts were named Pt-Ti-P (unirradiated TiO_2_) and Pt-Ti-P-x (x = 100, 300, 500, and 700).

#### 3.2.3. Methods for PAE Loading

Pt-Ti-P-x was mixed with 84 μL DMP or DEMP in 30 mL of acetone solution, sealed with parafilm, and then placed in a magnetic mixer for 30 min, followed by drying at room temperature for 24 h.

### 3.3. Structural Characterization

A comprehensive array of advanced characterization techniques was employed to investigate the physicochemical properties of the materials. X-ray diffraction (XRD) patterns were recorded using a Bruker D8 Advance diffractometer with Cu Kα radiation, covering the 2θ range of 20–80° at a scanning rate of 10°/min. X-ray photoelectron spectroscopy (XPS) analyses were conducted on an SC ALAB 250Xi spectrometer (Thermo Fisher Scientific, Waltham, MA, USA) equipped with a monochromatic Al Kα source under ultra-high vacuum conditions (<10^−7^ Pa), providing detailed insights into electronic states and elemental compositions. Meanwhile, C 1s at 284.8 eV was applied for spectra calibration. Morphological and structural characterization were performed using transmission electron microscopy (TEM) and high-resolution transmission electron microscopy (HRTEM) on a Talos F200S G2 instrument (Thermo Fisher Scientific). Time-resolved photoluminescence (TRPL) decay spectra were acquired at an excitation wavelength of 365 nm using a PMT detector on a Hitachi F-7000 fluorescence spectrometer (Tokyo, Japan). Gas chromatography–mass spectrometry (GC-MS; 7890A GC/5675CMS, Agilent, Santa Clara, CA, USA) was utilized to identify intermediate products at reaction temperatures of 125, 175, 200, 250, and 300 °C. UV–visible diffuse reflectance spectroscopy (UV-vis DRS) measurements were conducted at ambient conditions using a Shimadzu UV-2600 spectrophotometer (Shimadzu, Kyoto, Japan) to assess optical properties. Electrochemical impedance spectroscopy (EIS) and transient photocurrent (IT) measurements were performed using a CHI660C electrochemical workstation (Shanghai Chenhua Instrument Company, Shanghai, China) to evaluate charge transfer characteristics. Furthermore, in situ diffuse reflectance infrared Fourier transform spectroscopy (DRIFTS) was carried out on a Nicolet IS50 spectrometer (Thermo Fisher Scientific, USA) to elucidate reaction mechanisms.

### 3.4. Catalytic Reaction

#### 3.4.1. Photocatalytic Experiments

The photocatalytic performance of the PAEs was systematically evaluated using Fourier transform infrared (FTIR) spectroscopy in a custom-fabricated reactor. Photocatalytic experiments involved pressing 50 mg of potassium bromide powder into the reactor to establish a baseline spectrum, followed by the addition of 20 mg of catalyst mixed with the potassium bromide powder for the catalytic reactions. The samples were pre-treated with an argon flow (15 mL/min) for 35 min at room temperature to remove adsorbed water and impurities. During the catalytic tests, the samples were exposed to an oxygen–argon mixture (35 mL/min) and pure argon (15 mL/min), with FTIR spectra collected in the wavenumber range of 3500–1000 cm^−1^. The photocatalytic performance was assessed by recording the FTIR spectra after irradiating the reactor with a xenon lamp for 10 min to ensure equilibrium. At this time, the temperature inside the reactor was 35 °C due to the photothermal effect.

#### 3.4.2. Photothermal Catalytic Experiments

The photothermal catalytic performance of the PAEs was systematically evaluated using Fourier transform infrared (FTIR) spectroscopy in a custom-fabricated reactor. The quality of the catalysts and the pre-treatment before the experiment were consistent with photocatalytic experiments. During the catalytic tests, the samples were exposed to an oxygen–argon mixture (35 mL/min) and pure argon (15 mL/min), with FTIR spectra collected in the wavenumber range of 3500–1000 cm^−1^. Photothermal catalytic performance was evaluated by heating the reactor and recording FTIR spectra at 35, 75, 100, 125, 175, 200, 250, and 300 °C, maintaining each temperature for 10 min to ensure equilibrium.

## 4. Conclusions

In conclusion, Pt-Ti-P-x catalysts were synthesized by the impregnation method and modified by a high-energy electron beam irradiation technique to construct highly efficient photothermal catalysts enriched with surface O_V_s. Different irradiation doses (100, 300, 500, and 700 kGy) of the high-energy electron beam showed that a suitable irradiation dose (300 kGy) helps to introduce abundant O_V_s in the surface structure of the catalyst. XRD, Raman, and TEM characterization confirmed that high-energy electron beam irradiation modulated the catalyst surface structure, causing lattice distortions and introducing defects. EPR characterization confirmed the introduced defects were O_V_s. XPS showed that Pt-Ti-P-300 had the highest adsorbed oxygen content, and its ratio of adsorbed oxygen to lattice oxygen was the largest. UV-vis DRS characterization showed that the electron beam irradiation of the constructed O_V_s promoted visible light absorption and broadened the light absorption range. IT and EIS characterization showed that the O_V_s promoted the production and separation of photogenerated carriers. In addition, the photocatalytically generated active species promoted the breakage and deep oxidation of DMP side chains, which facilitated catalysis at lower temperatures. Finally, possible photothermal degradation pathways were proposed in combination with in situ DRIFTS and GC-MS. This work provides important insights into the electron beam irradiation-mediated regulation of catalysts and the photothermal catalytic removal of PAEs in indoor environments.

## Figures and Tables

**Table 1:** Physicochemical parameters of the Pt-Ti-P and Pt-Ti-P-x catalysts.

Sample	S_BET_ (m^2^/g)	V_tato_l (cc/g)	D (nm)	XPS		
				PtO_x_/Pt_total_	O_ads_/O_latt_	Ti^3+^/Ti^4+^
Pt-Ti-P	52.0	0.23	2.5–10	0.96	0.186	0.15
Pt-Ti-P-100	55.9	0.18	2.5–9.6	0.96	0.193	0.18
Pt-Ti-P-300	51.9	0.42	2.5–9.3	0.99	0.233	0.22
Pt-Ti-P-500	54.2	0.39	2.5–9.5	0.98	0.205	0.18
Pt-Ti-P-700	55.4	0.45	2.5–10	0.97	0.199	0.17

**Table 2:** Intermediate products of the photothermal catalytic degradation of DMP on Pt-Ti-P-300 detected by GC-MS.

Number	Time (min)	Compound Name	Molecular Formula
(**1**)	5.2	Acetone	C_3_H_6_O
(**2**)	6.2	Hexane	C_6_H_14_
(**3**)	9.0	Benzoic acid	C_7_H_6_O_2_
(**4**)	9.8	Toluene	C_7_H_8_
(**5**)	10.8	Benzene	C_6_H_6_
(**6**)	12.0	4-Methyl-3-penten-2-one	C_6_H_10_O
(**7**)	15.0	O-Xylene	C_8_H_10_
(**8**)	15.5	Paraxylene	C_8_H_10_
(**9**)	25.5	Phthalic anhydride	C_8_H_4_O_3_
(**10**)	29.6	Phthalic acid	C_8_H_6_O_4_
(**11**)	33.8	Methyl benzoate	C_8_H_8_O_2_
(**12**)	36.0	Dimethyl phthalate	C_10_H_10_O_4_
(**13**)	37.0	Dimethyl 5-methylisophthalate	C_11_H_12_O_4_

## Data Availability

The authors declare that all data supporting the findings of this study are available within the article.
